# Positional and between quarter differences in physical demands of elite female field hockey players during international matches

**DOI:** 10.3389/fspor.2023.1296752

**Published:** 2023-12-22

**Authors:** Colin Powell, Martinique Sparks, Cindy Pienaar

**Affiliations:** ^1^ Physical Activity, Sport and Recreation (PhASRec), North-West University, Potchefstroom Campus, Potchefstroom, South Africa; ^2^ Department of Sport Studies, Faculty of Applied Sciences, Durban University of Technology, Durban, South Africa

**Keywords:** relative load, Global Positioning System, activity profile, player load, hockey, fatigue, pacing

## Abstract

**Objectives:**

The main aims of this study were to examine whether there are positional- and between-quarter differences in the physical load of elite female field hockey players during international matches.

**Methods:**

Twenty-three international female field hockey players were equipped with Global Positioning Systems devices, while competing over nine international matches.

**Results:**

Players covered a mean relative distance, relative player load, and distance covered in the form of low-, moderate-, and high intensity activities of 107.5 m/min, 10.3 AU/min, 41.6%, 47.9%, and 9.9%, respectively. Defenders achieved the lowest relative Player load (ES: 0.8–1.1) and greatest distance covered in the form of low intensity activities compared to Midfielders and Forwards (ES: 0.8–0.9). Forwards and Midfielders covered significantly greater distance in the form of high intensity activity compared to Defenders (ES: 1.6–2.2). Significant reductions in relative distance, relative Player load, and moderate intensity activity are observed for all positions between Quarters 1–4 despite the availability of unlimited substitutions. However, players were able to maintain their high intensity activity throughout the match with no significant differences between Quarters 1–4. The majority of variables were higher (ES > 0.2) during Quarter 2 compared to Quarter 3, especially for Midfielders and Defenders.

**Conclusions:**

Current findings provide further evidence on the positional- and between-quarter external match load of international female field hockey players that coaches should consider when designing training programs and drills to better prepare players for match demands. The results also provide some insight into the fatigue experienced by players and the possible pacing strategies they employ during matches. These findings support the need for re-warm-ups and may further influence how coaches time their substitutions to enhance running performance in future.

## 1. Introduction

Field hockey is a popular team sport, played by both male and female competitors, with various competitions (i.e., World Cup, World League, Commonwealth Games etc.) catering for all ages and competitive standards (1–3). The evolution and professionalization of field hockey see matches played much differently compared to past decades, with multiple rule changes introduced, specifically to help increase the intensity of match-play (4–6). This includes, in 2015, a change in format of all international field hockey matches from playing two, 35 min halves, to four, 15 min quarters (Qtrs) (7–9). As a result, players' activity profile should be re-examined with differences in external (i.e., distance covered etc.) match loads reported (2, 3, 7, 9–11). These differences may lead to a restructuring of training prescriptions to better prepare players for the demands of match-play. However, due to the scarcity of research available, particularly on the positional and between-quarter match demands of elite female field hockey players post-format change, more research is required to broaden our understanding of the player's activity profile.

Despite a reduction in match duration from 70- to 60 min, research investigating the activity profile of international female field hockey players report near-identical distances covered between formats (2, 8–11). Matches played in the current format are also marked by greater intensities with larger reported percentages of match distance covered in the form of high-intensity activities (>4.5 m/s) (2, 8–10, 12). It is suggested that relative load, which is the normalization of distance covered to playing time (m/min), be examined to confirm whether matches under the current format are played at higher intensities. This is especially true because rolling substitutions and shorter match durations may affect the distance that players can cover (8, 9). Additional measures of relative PlayerLoadTM (RPL), which describe the activity profile through calculations of various locomotor activities (i.e., accelerations, changes of direction) could further expand our understanding of match load as opposed to measures of running distance alone (13–16).

Research examining physical demands report greater relative distances (RD) (128–147 m/min) covered under the current format compared to the previous (109–128 m/min), while RPL (measured in arbitrary units [AU] remained consistent (11.2–11.3 AU/min), indicating similar movement patterns (8–10, 17). Despite greater RD, the ability to sustain HIA appears diminished with reductions reported from Qtr 1 to Qtr 4 by Kapteijns et al. (10), possibly indicating increased fatigue, however in the earlier study by McGuinness et al. (2) they found an increase in HIA from Qtr 3 to 4. Although findings support the notion that the format change has resulted in an increase in match intensity, there is some disagreement on whether this intensity is maintained throughout the match, which necessitates further investigation (10, 18, 19). As a result, the main aims of this study were to examine whether there are positional and between-Qtr differences in the physical demands of elite female field hockey players under the current format.

## 2. Materials and methods

### 2.1. Participants

The study consisted of 23 elite female field hockey players (age: 28.4 ± 3.3 years, height: 165.7 ± 5.2 cm, body mass: 61.3 ± 4.6 kg) competing over nine international matches. Players were categorized according to position, namely forwards (Fwd), midfielders (Mid) and defenders (Def). Before data collection, ethical approval was obtained from the Health Research Ethics Committee, where the study was conducted (NWU-00965-19-A1-01). The research was conducted according to the Declaration of Helsinki. Participation in the study was voluntary and participants could withdraw at any time and without prejudice.

### 2.2. Match performance analysis

The activity profile of all players (starting line-up and bench) was recorded during match-play. However, match load recorded during benching periods was excluded from the analysis. Goalkeepers were also excluded from the analysis. Activities were measured with the Catapult MinimaxX S4 10-Hz Global Positioning Systems (GPS), equipped with a 100-Hz accelerometer (Catapult Innovations, Melbourne, Australia). The validity and reliability of these devices was previously verified (20–22). The GPS units were turned on and left idle for 10 min before pre-match warm-ups to allow for the location of satellites. After confirmation of satellite connection, the GPS units were fitted to the upper back of each player using a harness supplied by the manufacturer.

For statistical analysis, locomotor categories used were adapted from those previously used for female field hockey players, namely low- (LIA) (0–2.2 m/s); moderate- (MIA) (2.3–4.4 m/s); and HIA (≥4.5 m/s) (2, 8, 17). For a movement to be recorded as an effort, players had to maintain specific movement velocities for at least 0.5 s. PlayerLoad™ is a Catapult Innovations software measurement that instantaneous rate of change of acceleration divided by a scaling factor. Playerload gives an unbiased assessment of the athlete's workload at any given time by measuring the workload of players based on accelerations rather than distance. Player Load is the sum of the accelerations across all axes (anteroposterior, mediolateral and vertical acceleration) of the internal tri-axial accelerometer during movement. This metric is measured in arbitrary units. Numerous investigations looked into the validity and reliability of the Playerload metric and concluded that it has convergent validity with measures of exercise intensity in addition to acceptable within and between device reliability (13, 14, 23). All data were normalized to RD (m/min), RPL (AU/min) and percentage distance covered in LIA, MIA and HIA zones. The raw data were further divided into Qtrs 1–4. Additionally, horizontal dilution of precision (HDOP) and satellite availability were analyzed to ensure accurate measures of GPS devices in a horizontal plane. The mean HDOP and satellite availability was 0.93 ± 0.05 and 10.9 ± 0.4, respectively. GPS Doppler data was used during the analysis of the GPS-related variables after an intelligent motion filter was applied.

### 2.3. Statistical procedures

The Statistical Package for Social Sciences (IBM SPSS® Statistics version 28) was used for statistical analyses. Descriptive statistics [mean and standard deviation (SD)] were calculated and used to describe the external load of players throughout Qtrs 1–4. A one-way analysis of variance (ANOVA) was applied to measure potential differences in match load between positional groups for each Qtr. Another ANOVA was then applied to measure potential differences in match loads between Qtrs for each positional group. The level of statistical significance (*p*-value) was set at ≤0.05. When significant main effects were observed, the Bonferroni post-hoc test was used to determine where significant positional and between-Qtr differences lay. Cohen's D was used to determine the magnitudes of the standardized effect sizes [with 95% confidence intervals (CI)] were defined as: 0–0.2 trivial; 0.2–0.6 small; 0.6–1.2 moderate; 1.2–2.0 large; and >2.0 very large (24). A effect size (ES) greater than 0.2 was seen as worthwhile, however, if the lower and upper CI exceeded negative (−0.2) and positive (0.2) values, the difference was deemed unclear, and no inference was made on whether “true” differences could be observed in the greater population (24).

## 3. Results

Descriptive data (mean ± SD) on external load for players are presented in Table 1. The mean RD, RPL, LIA-, MIA-, and HIA% covered during match-play were 107.5 m/min, 10.3 AU/min, 41.6%, 47.9%, and 9.9%, respectively. Def compared to Mid and Fwd achieved the lowest RPL (ES: 0.8–1.1) and greatest distance covered in the form of LIA (ES: 0.8–0.9). Fwd and Mid compared to Def (ES: 1.6–2.2) covered significantly greater distance in the form of HIA.

**Table 1:** Descriptive data (mean ± SD) of external match load for international female field hockey players.

Variable	Period	Total	Fwd	Mid	Def
		(*n* = 142)	(*n* = 47)	(*n* = 48)	(*n* = 47)
RD (m/min)	Qtr 1	114.1 ± 14.8	114.5 ± 10.6	113.7 ± 17.6	114.2 ± 15.5
	Qtr 2	107.7 ± 17.1	103.9 ± 19.2	110.6 ± 18.6	108.4 ± 12.4
	Qtr 3	105.3 ± 19.2	103.9 ± 23.3	106.7 ± 18.4	105.3 ± 15.5
	Qtr 4	103.6 ± 14.9	102.9 ± 16.6	105.9 ± 15.4	102.0 ± 12.5
	TOTAL	107.5 ± 12.6	106.3 ± 14.3	109.0 ± 11.5	107.1 ± 12.0
RPL (AU/min)	Qtr 1	11.0 ± 1.6	11.8 ± 1.3^b,c^	10.9 ± 1.6	10.3 ± 1.5
	Qtr 2	10.4 ± 1.9	10.9 ± 2.2^c^	10.7 ± 2.0^c^	9.7 ± 1.4
	Qtr 3	10.0 ± 2.0	10.6 ± 2.6^c^	10.2 ± 1.8^c^	9.2 ± 1.3
	Qtr 4	9.9 ± 1.6	10.6 ± 1.7^c^	10.2 ± 1.6^c^	8.9 ± 1.0
	TOTAL	10.3 ± 1.4	11.0 ± 1.5^c^	10.4 ± 1.2^c^	9.5 ± 1.1
LIA%	Qtr 1	39.7 ± 5.6	38.3 ± 6.1	39.7 ± 5.9	41.0 ± 4.5^a^
	Qtr 2	40.9 ± 5.8	40.6 ± 6.3	39.4 ± 5.5	42.9 ± 4.9^b^
	Qtr 3	43.2 ± 8.4	42.2 ± 11.2	42.3 ± 7.2	45.0 ± 6.1
	Qtr 4	42.7 ± 7.7	40.8 ± 9.5	41.6 ± 6.7	45.6 ± 5.7^a,b^
	TOTAL	41.6 ± 4.5	40.1 ± 4.9	40.4 ± 3.6	43.8 ± 4.2^a,b^
MIA%	Qtr 1	50.3 ± 5.5	50.8 ± 5.5	48.7 ± 5.4	51.4 ± 4.4^b^
	Qtr 2	48.2 ± 5.6	47.9 ± 5.9	47.9 ± 6.1	48.7 ± 4.7
	Qtr 3	46.8 ± 7.6	46.1 ± 9.3	46.4 ± 7.1	47.9 ± 6.0
	Qtr 4	45.6 ± 7.6	46.6 ± 9.3	44.9 ± 7.3	45.5 ± 6.2
	TOTAL	47.9 ± 3.9	48.3 ± 3.7	47.0 ± 4.1	48.4 ± 3.9
HIA%	Qtr 1	9.1 ± 3.7	9.9 ± 3.7^c^	10.7 ± 3.5^c^	6.5 ± 2.6
	Qtr 2	10.0 ± 4.3	10.6 ± 4.3^c^	11.9 ± 4.5^c^	7.4 ± 2.6
	Qtr 3	9.2 ± 4.4	10.8 ± 4.6^c^	10.4 ± 4.1^c^	6.3 ± 3.0
	Qtr 4	10.2 ± 4.4	9.7 ± 4.4	12.8 ± 4.4^a,c^	7.9 ± 2.8
	TOTAL	9.9 ± 3.0	10.7 ± 2.7^c^	11.8 ± 2.4^c^	7.2 ± 1.6

RD, Relative distance (m/min); RPL, Relative player load (AU/min); LIA-, MIA-, HIA%, percentage of distance covered at low-, moderate-, and high intensity; Qtr, quarter.

Subscript.

^a^ Significant difference compared to Fwd.

^b^ Significant difference compared to Mid.

^c^ Significant difference compared to Def.

**Figure 1:**
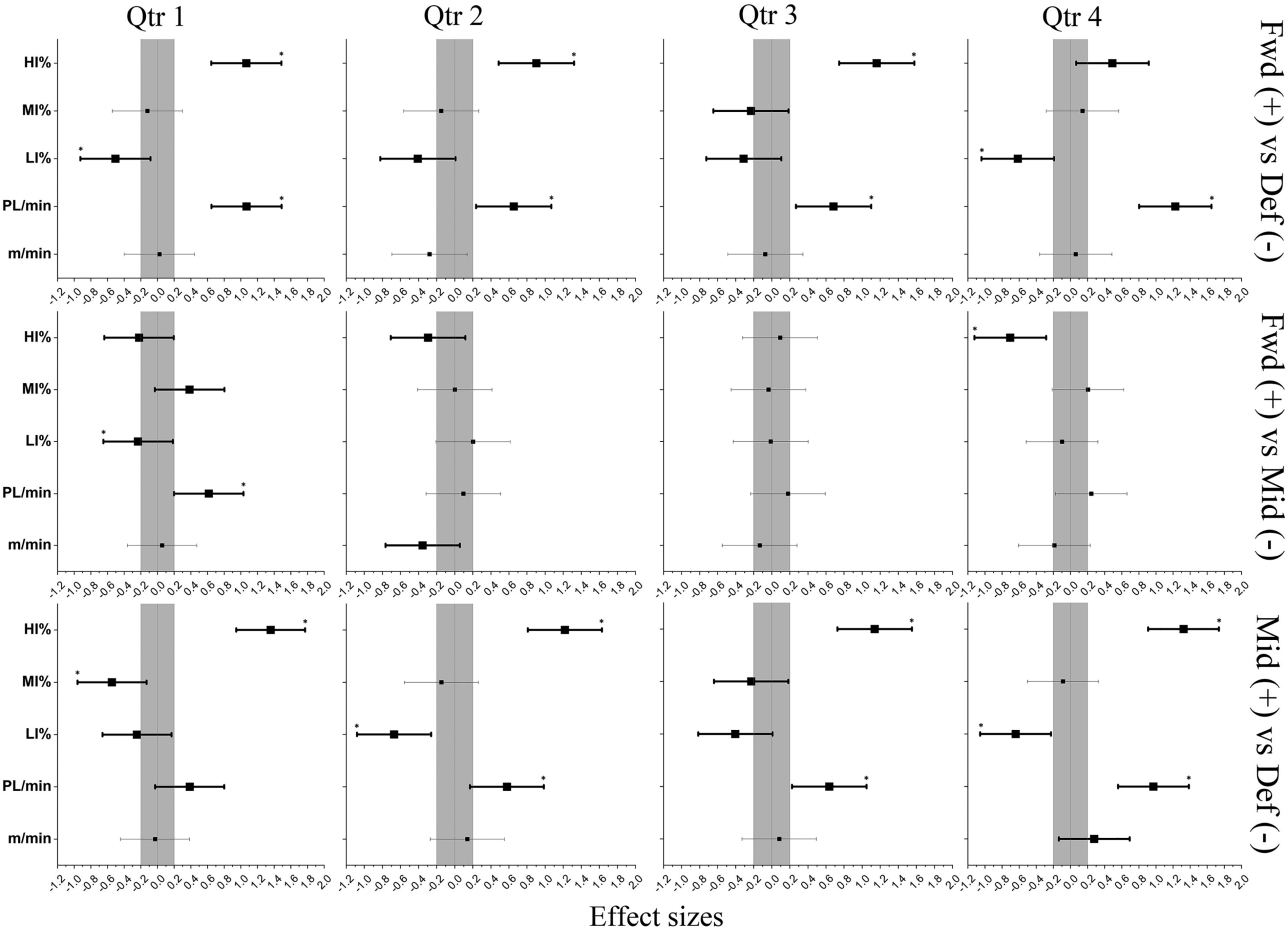
Positional differences in external match load for each quarter of match-play. *: Statistically significant difference between quarters. The darker shaded lines represent meaningful inferences for ES and 95% CI.

**Figure 2:**
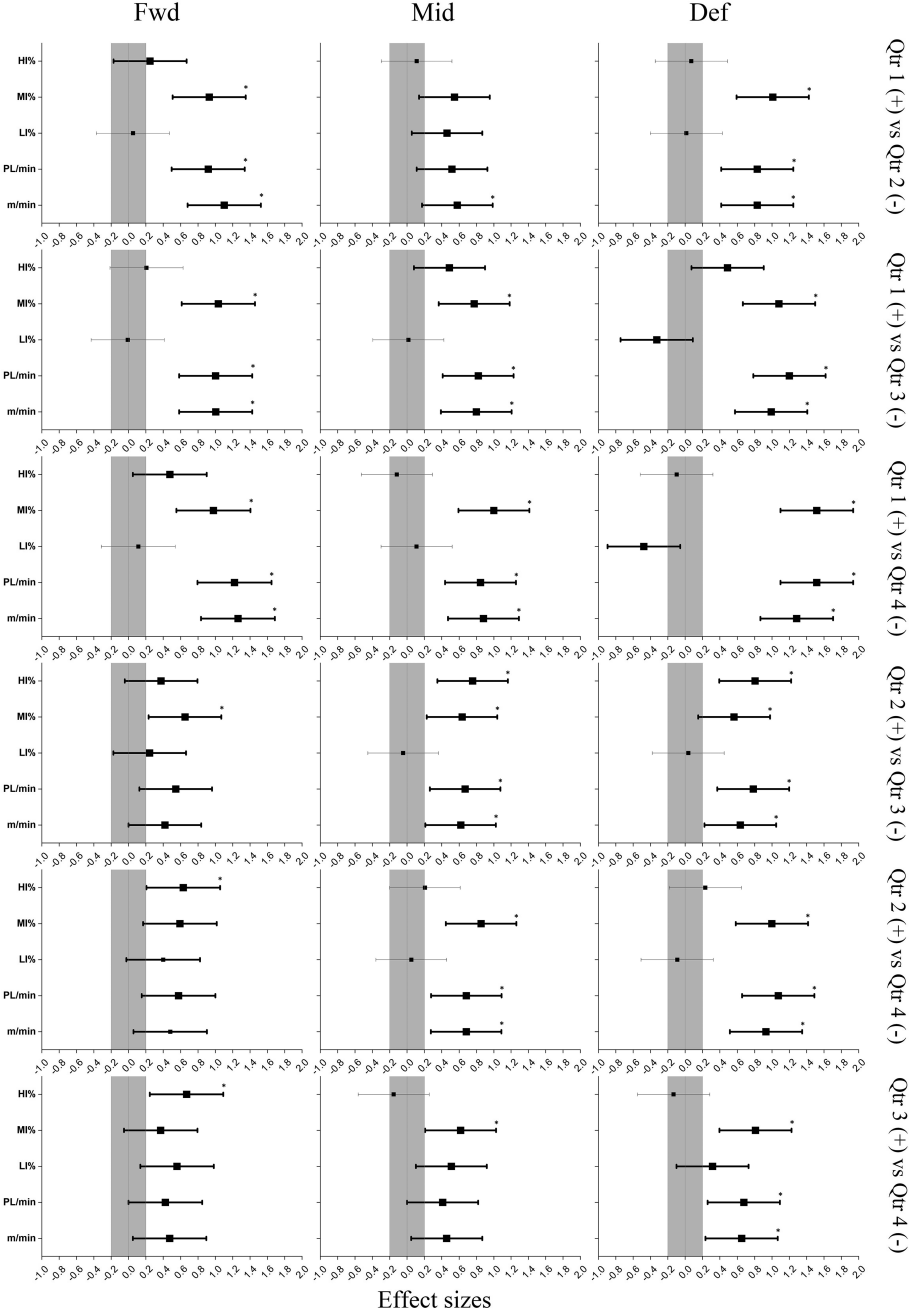
Differences in external match load between quarters by playing position. *: Statistically significant difference between quarters. The darker shaded lines represent meaningful inferences for ES and 95% CI.

Positional and between-Qtr differences in external load are presented in Figures 1, 2, respectively. The *p*-value indicated by “*” shows that significant differences existed among positional groups in terms of distances covered in the form of LIA, MIA, and HIA, as well as RPL. The darker shaded lines represent meaningful inferences for ES. Throughout Qtrs 1–4, Fwd attained significantly greater RPL compared to Def (ES: 0.6–1.2), along with greater RPL compared to Mid during Qtr 1 (ES: 0.6). Mid attained a significantly greater RPL (ES: 0.6–1.0) compared to Def during Qtrs 2–4. During Qtrs 2 and 4, Def covered significantly greater distances in the form of LIA compared to Mid (ES: 0.6–0.7), with greater distances covered compared to Fwd during Qtrs 1 and 4 (ES: 0.5–0.6). Furthermore, Def covered significantly greater distances in the form of MIA compared to Mid during Qtr 1 (ES:0.6). Throughout Qtrs 1–4, Mid covered significantly greater distances in the form of HIA (ES: 0.9–1.3) compared to Def, with greater distances covered compared to Fwd during Qtr 4 (ES: 0.7). Fwd covered greater distances in the form of HIA compared to Def (ES: 0.9–1.2) during Qtrs 1–3.

In terms of between-Qtr differences, RD was significantly greater among all positions during Qtr 1 compared to Qtrs 2–4 (ES: 0.6–1.2). RD for Mid and Def during Qtr 2 compared to Qtrs 3–4 (ES: 0.6–0.9) was significantly greater. Def achieved significantly greater RD during Qtr 3 compared to Qtr 4 (ES:0.6). During Qtr 1 compared to the other Qtrs, all positional groups achieved significantly greater RPL (ES: 0.8–1.5). During Qtr 2, RPL was significantly greater for Mid, and Def compared to Qtrs 3–4 (ES: 0.6–1.1). RPL was also significantly greater for Def during Qtr 3 compared to Qtr 4 (ES: 0.6). With regards to distances covered at different intensity zones, MIA was significantly greater for all positional groups during Qtr 1 compared to Qtrs 2–4 (ES: 0.8–1.5). During Qtr 2 compared to Qtr 3 (ES: 0.6), MIA was significantly greater for Fwd and Mid. MIA was greater for Mid and Def during Qtrs 2 and 3 compared to Qtr 4 (ES: 0.6–0.8). Finally, during Qtr 2 compared to Qtr 3 (ES: 0.7–0.8) HIA was significantly greater for Mid and Def. HIA was also significantly greater for Fwd during Qtrs 2–3 compared to Qtr 4.

## 4. Discussion

The main aims of this study were to examine whether there are positional and between-Qtr differences in the physical demands of elite female field hockey players during match play. Research concerning positional and between-Qtr differences in match load under the current format is limited. Therefore, an examination of the activity profile under the current format is necessitated to better understand current match demands, which will assist coaches to better prepare players for the demands of competition. The main finding of this study shows that positional differences in external match load exist for international female field hockey players. Findings also show significant between-Qtr differences for various external loads between all positions, despite the availability of unlimited substitutions and less match time.

The current study show that players cover RD of 107.5 m/min during match-play, which is similar to those previously reported for international female players (113 m/min) (9). Although greater RD (128–147 m/min) have been reported elsewhere (2, 10, 11), lower RD observed in the current study could be attributed to greater distances covered in the form of LIA and MIA. Our study shows that players achieved RPL of 10.3 AU/min, which is similar than those previously reported for international female field hockey players (11.2 AU/min) (9). In agreement with previous findings our study showed that Fwd (11.0 AU/min) and Mid (10.4 AU/min) achieved significantly greater RPL throughout match-play compared to Def (9.5 AU/min) (9). Defenders having a lower work-rate and intensity compared to Fwd and Mid might be due to the tactical defensive structure of the specific team. They employed a zonal marking system in stead of a one-to-one marking system. Konarski (25) established that the workload and intensity during a man to man marking system is considerably higher than when teams employ a zonal marking system. This is further supported by the data showing that Def tended to spend more time in LIA and MIA compared to the other positional groups. In contrast to Morencos et al. (11) who found that Fwd had more HIA compared to Mid, our study indicated that Mid had more HIA compared to Fwd. Again, these results could be due to differences in team tactics and might even indicate differences between men and female hockey match play. The study by Morencos et al. (11) was done on international men hockey players, whereas our study was conducted on international female hockey.

When the ability of players to sustain higher intensities throughout the match is considered, significant reductions in RD, RPL, and MIA are observed for all positions between Qtrs 1–4 despite the availability of unlimited substitutions. However, players were able to maintain their HIA throughout the match with no significant differences between Qtrs 1–4. These findings are in agreement with Morencos et al. (11) who also found significant declines in RD but with no significant declines in HIA in male hockey. Ihsan et al. (7) suggested in this regard that players might use pacing strategies throughout the match by maintaining their HIA and altering their lower intensity activities. Recent results from McGuiness et al. (8) support this notion of pacing in field hockey with further evidence provided in football (26, 27). Even though the reduction in work rate suggests that the accumulation of fatigue is present, despite unlimited substitutions, the players were able to some extent mitigate reductions in intensity with the use of substitutions and possible pacing. Interestingly, the majority of external demand variables for were higher during Qtr 2 compared to Qtr 3, especially for Mid and Def. These results are interesting considering that players have a prolonged break (10 min) between Qtr 2 and Qtr 3 and one would expect sufficient time for players to recover. In a recent systematic review by Gonzales-Devesa et al. (28), concluded that several studies have found a decline in physical and cognitive performance after half-time breaks and supported the notion of re-warm-ups. This was further supported by Christaras et al. (29) who found that a re-warm-up protocol improved the jump and sprint performance of youth football players. However, due to the low number of matches analyzed the authors suggested improvements in running performance could not be proven and needs some further investigation (29).

In summary, Def in this study had significantly lower work rates and intensities with Mid having performed more strenuous activities compared to the other positional groups. When between-Qtr differences in external match load are considered, data from the current study show significant declines in work rate from Qtr 1–4, however players were able to maintain the intensity throughout the match indicating possible pacing strategies. Significant declines in performance were also seen between Qtr 2 and 3. Although more evidence is needed, it can be suggested that there is a possible need for re-warm-ups after the prolonged break after Qtr 2 and warrants further investigation. The findings of this study, however, should be regarded with caution because they reflect the external demands of only one team. Even though the opponents faced were both higher and lower ranked than the investigated team, team strategies, strengths, and weaknesses may influence how they play the match and the resultant impact on external player loads. Also, combining movement data with video-based statistics will give greater technical and tactical insights into different phases of play (i.e., transition/ attacking/ defensive phases, leading/ trailing etc.) and should be explored in future studies. Nonetheless, the results from this study provide an improved understanding of the external demands players face during international matches and indicate the need for position specific conditioning plans. The results also provide some insight into the fatigue experienced by players and the possible pacing strategies they employ during matches. These findings support the need for re-warm-ups and may further influence how coaches time their substitutions to enhance running performance in future.

## Data Availability

The raw data supporting the conclusions of this article will be made available by the authors, without undue reservation.
